# Supplementary material for: The Characteristics of Cognitive Proficiency in Patients with Acute Neuromyelitis Optica Spectrum Disease and its Correlation with Serum Aquaporin-4 Antibody Titer
Source: Brain Sci. 2023 Jan 2;13(1):90. doi: 10.3390/brainsci13010090 (PMC9857218; doi:10.3390/brainsci13010090)
Supplement: Supplementary file 1 [file brainsci-13-00090-s001.zip › brainsci-2086175-supplementary.pdf]

Table S1. The correlation between age and cognitive evaluation scores

cognitive evaluation scale	Age			
	Kendall			Spearman
	R	P	R	P
**Corrected MoCA**	-0.358	0.015*	-0.5	0.011*
**CR-AVLT-N5**	-0.199	0.173	-0.306	0.137
**CRAVLT-N6**	-0.257	0.094	-0.341	0.096
**CRAVLT-N7**	-0.056	0.706	-0.080	0.704
**CRAVLT-N8**	0.096	0.522	-0.139	0.507
**CRAVLT-N9**	0.007	0.962	0.018	0.932
**ROCF-C**	0.058	0.708	0.101	0.631
**ROCF-R**	0.020	0.888	0.058	0.781
**PASAT-3**	-0.174	0.231	-0.245	0.237
**PASAT-2**	-0.164	0.260	-0.236	0.256
**CWT-A**	0.173	0.232	0.265	0.200
**CWT-B**	0.112	0.440	0.163	0.436
**CWT-C**	0.166	0.251	0.225	0.280
**DST**	-0.257	0.092	-0.344	0.092
**VFT**	-0.281	0.054	-0.387	0.056

*P＜0.05.

Table S2. The correlation between years of education and cognitive evaluation scores

**cognitive evaluation scale**	**Years of Education**			
	Kendall			Spearman
	R	P	R	P
**Corrected MoCA**	0.469	0.003**	0.574	0.003**
**CR-AVLT-N5**	0.232	0.138	0.297	0.150
**CRAVLT-N6**	0.508	0.002**	0.607	0.001**
**CRAVLT-N7**	0.218	0.172	0.266	0.199
**CRAVLT-N8**	0.204	0.203	0.249	0.231
**CRAVLT-N9**	0.280	0.088	0.380	0.061
**ROCF-C**	0.218	0.189	0.267	0.196
**ROCF-R**	0.219	0.159	0.320	0.118
**PASAT-3**	0.292	0.062	0.384	0.058
**PASAT-2**	0.436	0.005**	0.573	0.003**
**CWT-A**	-0.349	0.024*	-0.458	0.021*
**CWT-B**	-0.349	0.024*	-0.433	0.031*
**CWT-C**	-0.146	0.345	-0.177	0.389
**DST**	0.378	0.020*	0.466	0.019*
**VFT**	0.293	0.061	0.391	0.053

***P＜0.05. **P＜0.01.**

Table S3. The correlation between intracranial lesions and cognitive evaluation scores

cognitive evaluation scale	Intracranial lesions			
	Kendall		Spearman	
	R	P	R	P
**Corrected MoCA**	-0.435	0.012*	-0.511	0.009**
**CR-AVLT-N5**	-0.196	0.255	-0.233	0.263
**CRAVLT-N6**	-0.297	0.101	-0.335	0.102
**CRAVLT-N7**	-0.249	0.155	-0.290	0.159
**CRAVLT-N8**	-0.163	0.355	-0.189	0.367
**CRAVLT-N9**	-0.138	0.445	-0.156	0.457
**ROCF-C**	-0.312	0.088	-0.348	0.088
**ROCF-R**	-0.385	0.025*	-0.459	0.021*
**PASAT-3**	-0.167	0.331	-0.198	0.342
**PASAT-2**	-0.238	0.165	-0.283	0.170
**CWT-A**	0.415	0.015*	0.498	0.011*
**CWT-B**	0.273	0.108	0.328	0.109
**CWT-C**	0.302	0.076	0.362	0.075
**DST**	-0.133	0.459	-0.151	0.471
**VFT**	-0.172	0.317	-0.204	0.328

***P＜0.05. **P＜0.01.**
